# Biomaterial‐Based Therapeutic Delivery of Immune Cells

**DOI:** 10.1002/adhm.202400586

**Published:** 2024-06-05

**Authors:** Ameya A. Dravid, Ankur Singh, Andrés J. García

**Affiliations:** ^1^ Woodruff School of Mechanical Engineering Georgia Institute of Technology Atlanta GA 30332 USA; ^2^ Coulter Department of Biomedical Engineering Georgia Institute of Technology and Emory University Atlanta GA 30332 USA; ^3^ Petit Institute for Bioengineering and Bioscience Georgia Institute of Technology Atlanta GA 30332 USA

**Keywords:** biomaterials, cancers, cell therapies, diabetes, hydrogels, inflammatory diseases

## Abstract

Immune cell therapy (ICT) is a transformative approach used to treat a wide range of diseases including type 1 diabetes, sickle cell disease, disorders of the hematopoietic system, and certain forms of cancers. Despite excellent clinical successes, the scope of adoptively transferred immune cells is limited because of toxicities like cytokine release syndrome and immune effector cell‐associated neurotoxicity in patients. Furthermore, reports suggest that such treatment can impact major organ systems including cardiac, renal, pulmonary, and hepatic systems in the long term. Additionally, adoptively transferred immune cells cannot achieve significant penetration into solid tissues, thus limiting their therapeutic potential. Recent studies suggest that biomaterial‐assisted delivery of immune cells can address these challenges by reducing toxicity, improving localization, and maintaining desired phenotypes to eventually regain tissue function. In this review, recent efforts in the field of biomaterial‐based immune cell delivery for the treatment of diseases, their pros and cons, and where these approaches stand in terms of clinical treatment are highlighted.

## Graphical abstract

Adoptive transfer of immune cells is an effective approach to treat refractory diseases. However, clinical reports investigating this strategy demonstrate limitations like lack of localization of cells and modest efficacy. Recent efforts in engineering cell carriers have addressed these challenges, thus improving outcomes in vivo. This review highlights recent efforts to engineer immune cell carriers and their impact on treatment.

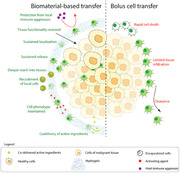

## 1 Introduction

### 1.1 Origin and Need of Cell Therapy

Common therapeutic modalities rely on the administration of small‐molecule drugs to treat diseases. However, small molecule drugs often suffer from off‐target effects, toxicity, low bioavailability, and rapid clearance. Recently, biologics (like Altuviiio and RabAvert for treating hemophilia and rabies, respectively^[^ ^1^ ^]^) have emerged as promising treatment alternatives because of their specificity and potency. However, these drugs (mostly delivered parenterally) exhibit suboptimal pharmacokinetics,^[^ ^2^, ^3^ ^]^ thus limiting their clinical application. Additionally, both modalities require repeated dosing to maintain therapeutically acceptable concentrations. Gene therapy has emerged as a viable alternative to conventional treatments. However, many gene vectors like engineered adeno‐associated viruses are inactivated by the immune system rapidly; repeating with larger doses only results in faster clearance and is not a viable solution for many patients.^[^ ^4^ ^]^ Vectors that achieve payload delivery run the risk of integrating genes of interest at unintended sites which can lead to severe adverse events.^[^ ^5^, ^6^ ^]^

**Figure 1:**
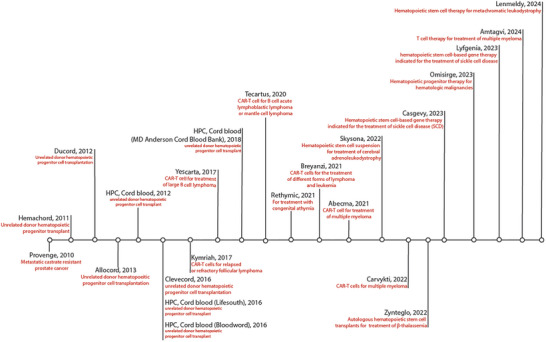
Timeline of immune cell therapies approved by the FDA to date.

To address these limitations, strategies that generate sufficient and durable efficacy need to be devised. Cell therapy aims to tune innate cellular functions to achieve specific, targeted treatment while maintaining efficacy for prolonged duration. Engineering therapeutic cells to facilitate the expression of poorly expressed/ non‐native proteins widens the functional scope, thus improving therapy compared to non‐engineered cells. The first personalized cancer vaccine approved by the US Food and Drug Administration (FDA), PROVENGE (Sipoleucel‐T), was a treatment that administered autologous engineered antigen‐presenting cells to treat prostatic acid phosphatase(PAP)^+^ prostate cancers.^[^ ^7^ ^]^ PROVENGE eventually failed (reviewed elsewhere^[^ ^8^ ^]^), but it opened avenues for the treatment of diseases using immune cell therapy (ICT). Consequently, in 2017, engineered T cell therapy (Chimeric Antigen Receptor T cells; CAR‐T) was approved by the FDA for the treatment of acute lymphoblastic leukemia in children.^[^ ^9^, ^10^ ^]^ Since then, the FDA has approved 6 CAR‐T cell products against various cancers (reviewed elsewhere^[^ ^11^ ^]^). All ICTs approved by the FDA for human use are summarized in **Figure** **1**.

### 1.2 Cell Therapy Offers Several Advantages over Current Treatment Modalities

- Safety: Currently approved immune cell therapy (CAR‐T cells) derives the base T cells from the patient being treated.^[^ ^12^ ^]^ Owing to the autologous origin of the cells, this treatment does not trigger a host‐versus‐graft immune response (however the treatment can cause toxicity in excessive doses). Several clinicians observe that the overall health of CAR‐T cell‐treated patients is recovered much faster compared to chemotherapy patients.
- Specificity: Immune cells can be engineered to identify and target specific markers. For example, current CAR‐T cells specifically neutralize CD19^+^ or B‐cell maturation antigen expressing cells. Similarly, clinical trials for Natural Killer (NK) cells targeting CD19 are also underway.^[^ ^13^ ^]^ Although certain stimuli‐responsive drug delivery vehicles claim specificity of interaction as well, such technologies face several challenges on the road to translation, with several of them failing to fulfill their primary criteria in clinical trials.^[^ ^14^ ^]^ Furthermore, with current genome editing capabilities, it is possible to confer a wide range of specificities to immune cells.^[^ ^15^, ^16^, ^17^ ^]^
- Wide variety of cells available for engineering and treatment: Various immune cells can be obtained from the host and engineered for therapeutic purposes. For instance, NK cells,^[^ ^18^ ^]^ T cells,^[^ ^19^ ^]^ and dendritic cells(DCs).^[^ ^20^ ^]^ have been explored for the treatment of glioblastoma. Similarly, for the treatment of type 1 diabetes, regulatory T cells and tolerogenic DCs have been evaluated.^[^ ^21^, ^22^ ^]^ Additionally, with the development of CRISPR‐Cas‐based gene editing techniques, it is possible to engineer cells against diverse specificities and molecular targets.^[^ ^15^, ^16^, ^17^, ^23^ ^]^
- Stimuli‐responsive treatment: The disease landscape changes very rapidly, with several markers being expressed only transiently. Such transient expression patterns can prevent recognition and successful targeting by the administered therapeutic cell. Recent reports demonstrate a design where engineered T cells express CAR only on contact with a tumor‐specific antigen.^[^ ^24^, ^25^ ^]^ The ability of engineered cells to identify cues from the environment and generate an appropriate, tuned response improves the specificity of treatment and also reduces off‐target toxicities.
- Shorter duration of treatment: ICT (especially CAR‐T cell therapy) has shorter durations of treatment (≈2,3 weeks) compared to standard of care like chemotherapy (which can potentially take months‐years).

### 1.3 Limitations of Cell Therapy

Findings from clinical trials indicate that adoptively transferred cells suffer from low in vivo persistence.^[^ ^26^ ^]^ Increasing the dose of administered CAR‐T cells is not feasible because it increases the chances of toxicity to vital tissues, cytokine release syndrome, tumor lysis syndrome, or fatal central nervous system‐related difficulties (detailed elsewhere^[^ ^27^ ^]^). These on‐target/off‐tumor effects have been attributed to a lack of localization of CAR‐T cells to the target. Additionally, the phenotype of transferred cells shifts to a non‐therapeutic state in some cases (e.g., CAR‐T cells adopt an exhausted phenotype^[^ ^28^ ^]^), thus dampening therapeutic response in vivo.^[^ ^29^ ^]^ This limitation can be attributed to the lack of sufficient exposure to activating cytokines. Despite the successful treatment of hematological tumors in human patients, treatment of solid tumors with CAR‐T cells is still challenging because of the restricted entry of cells into intratumoral sites and the immunosuppressive tumoral environment.^[^ ^30^ ^]^ Several of these challenges can be addressed by delivering cells via specially engineered biomaterials, as we will discuss below.

### 1.4 Overview of Challenges Solved by Biomaterial‐Based Approaches

**Figure 2:**
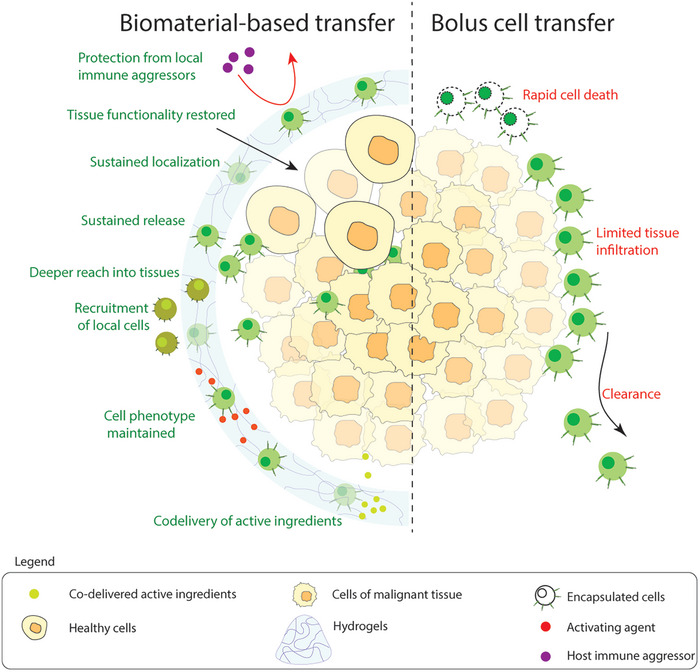
Advantages of using biomaterial‐based immune cell delivery compared to bolus transfer.

Current immune cell therapy faces two major challenges:^[^ ^1^ ^]^ insufficient penetration of therapeutic cells, and^[^ ^2^ ^]^ low numbers of therapeutic cells at the site of pathology to induce efficacy.^[^ ^31^, ^32^, ^33^ ^]^ A biomaterial‐based carrier can improve the localization of cells in the vicinity of affected tissue and act as a reservoir of therapeutic cells as the carrier is degraded. As a result, higher tissue penetration can be achieved.^[^ ^34^, ^35^, ^36^ ^]^ To further improve the scope of treatment, the co‐delivery of other active ingredients like cytokines that maintain activation, oncolytic viruses, and nanoparticles that improve the injectability of hydrogels have also been explored.^[^ ^35^, ^37^ ^]^ Solid tumors often have an immunosuppressive environment, which inactivates the administered CAR‐T cells. Biomaterials loaded with specific cytokines [like Interleukin (IL)‐15 and Interferon (IFN)‐*γ*] can help maintain the activation state longer, thus increasing the chances of successful treatment.^[^ ^35^, ^36^ ^]^ Overall, cells delivered via biomaterials have deeper reach into the tissue, have sustained localization at the site of disease, and maintain activated phenotype longer, thus improving the therapeutic efficacy compared to bolus‐delivered cells (**Figure** 2). As mentioned earlier, the constantly activated state of CAR‐T cells leads to limited efficacy due to the development of exhaustion phenotype. Recent studies indicate that hydrogels can be used to deliver therapeutics (like anti‐CTLA4 and anti‐TIGIT antibodies) which reverse the effects of exhaustion in CD8^+^ T cells.^[^ ^38^, ^39^ ^]^

### 1.5 Design Criteria for Delivery Vehicles

**Figure 3:**
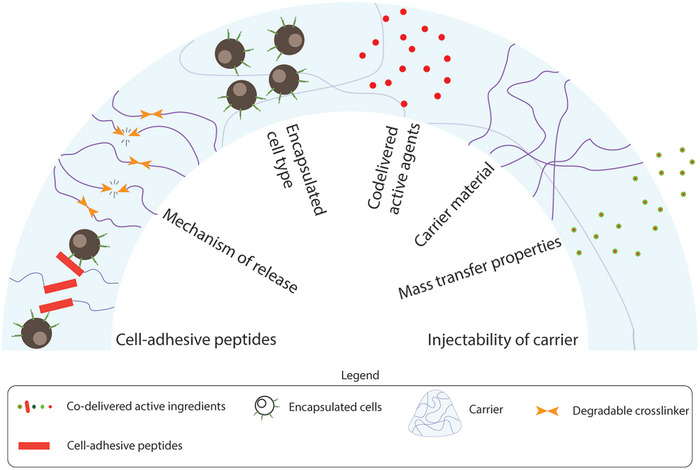
Parameters to consider while designing immune cell carriers: Carrier parameters mentioned above should be considered while designing treatment regimens.

Cell carriers are biomaterials that provide support, increase the persistence of cells at the site of delivery, and act as a sustained source of cells as the carriers degrade. The design of the cell carrier impacts cell phenotype, viability, integration with surrounding tissues, and mode of transfer. For cell delivery, the carrier should be engineered for several criteria (**Figure** 3):

1. Carrier material: The cell carrier should be cytocompatible with the encapsulated cells. Administered immune cells can either have a direct killing (CAR‐T cells), chemotactic, or regulatory effect on the surrounding environment. The material used to synthesize these carriers should be such that they either avoid interference with or enhance the efficacy. The majority of studies discussed in this review rely on hydrogels of synthetic polymers (like poly(ethylene glycol); PEG) for this purpose, but a few explore the use of biopolymers (like alginate and chitosan^[^ ^40^ ^]^). It is possible to leverage the strengths of different polymer types to synthesize suitable carriers, but such approaches are not popular in immune cell delivery. Carriers that need pre‐implantation steps (like mixing of engineered cells before delivery) should be resilient to commonly used sterilization techniques like gamma irradiation and ethylene oxide.
2. Cytokines and other bioactive factors: Carriers should support the viability of the cells and help maintain therapeutic phenotype. Several studies have demonstrated that a high local concentration of IL‐15 maintains the activated state of engineered T cells, thus providing longer‐lasting protection.^[^ ^34^, ^35^ ^]^ Similarly, the local presence of cytokines IL‐12 and Granulocyte‐macrophage colony‐stimulating factor (GM‐CSF) improves the survival of DCs in vivo.^[^ ^37^ ^]^ In addition to such cytokines, other factors that do not impact the encapsulated cells but supplement treatment (such as small molecule drugs^[^ ^41^ ^]^ and oncolytic viruses^[^ ^37^ ^]^) can also be included to improve outcomes. Other materials such as nanoparticles can also be added to the biomaterials to achieve the desired consistency, flow behavior, and injectability.^[^ ^35^ ^]^
3. Mass transfer capabilities: For the long‐term action and survival of cells in vivo, the carriers must allow the exchange of nutrition and molecules between the cells and their surroundings. To achieve this, it is important to optimize a suitable mesh size of the carrier.
4. Mechanism of release: The release of therapeutic cells from their carriers is crucial for their entry into diseased tissue. Typically, the mesh sizes of administered hydrogels range from 10–100 nm (depending on the swelling ratio and degree of crosslinking).^[^ ^42^ ^]^ Negligible cell release occurs passively by diffusion. To improve cell release kinetics, hydrogels can be crosslinked using stimuli‐response moieties that can be cleaved using external stimuli, such as secreted proteases,^[^ ^43^ ^]^ reactive oxygen species,^[^ ^44^ ^]^ and hydrolysis.^[^ ^45^ ^]^ The release kinetics of cells from hydrogels can be further impacted by the development of fibrous capsules at the gel implantation site. Introducing implants in an organism results in a foreign‐body response (reviewed in detail elsewhere^[^ ^46^ ^]^), characteristics of which are infiltration of cells like macrophages and fibroblasts, which ultimately results in the formation of a fibrous capsule around the implant, thus inhibiting cell release from the implant. Although absolute prevention of fibrous capsules has not yet been demonstrated, tethering PEG‐macromers with oligopeptide Arginine‐Glycine‐Aspartic acid (RGD) produced less dense fibrous capsules.^[^ ^47^ ^]^ The foreign body response can also be reduced by modulating properties of implanted gels like stiffness and hydrophilicity,^[^ ^48^ ^]^ where softer gels are reported to elicit milder responses. By limiting the foreign body response, it is possible to generate efflux of administered cells predictably.

## 2 Delivery of Lymphoid Cells

### 2.1 Delivery of T Cells Using Biomaterials

Engineered T cells are the most successful immune cells delivered therapeutically and have well‐established editing protocols. However, the restrictive entry of these cells into solid tissues has been a major limitation. Among other solid tissues, the brain offers added challenges because of the blood‐brain barrier (BBB; the tight layer of endothelial cells that separate the vasculature from the brain parenchyma). The convection‐enhanced delivery (CED) technology to deliver small‐molecule therapeutics with high efficiency in the brain, while bypassing the BBB, was developed in the 1990s.^[^ ^49^ ^]^ In CED, a special catheter is carefully localized in the brain which is used to “push” the active ingredient into the brain. Atik et al. observed that delivery of CAR‐T cells to the brain via CED is hindered because of the high sedimentation of cells in the catheter. To address this challenge, they developed a biocompatible, low‐viscosity hydrogel that maintains the CAR‐T cells in a suspension and prevents sedimentation in the device.^[^ ^34^ ^]^ In their experimental setup, the catheter‐delivering cells intracranially were connected to a syringe loaded with cells suspended in the low‐viscosity hydrogel. The syringe was driven by a syringe pump, which delivered cells to the brain in a continuous fashion over the next 5–7 h. The hydrogel prevented sedimentation of cells in the syringe and catheter, thus improving the efficiency of delivery compared to cells delivered in saline in the murine brain.^[^ ^34^ ^]^ This technique has strong potential and should be investigated further with further optimization of hydrogel synthesis and demonstration of long‐term efficacy.

Several reports have explored carriers that co‐delivered supplemental bioactive material (along with therapeutic cells) to improve the scope of the therapy, as we will discuss below. Chao et al. developed an alginate hydrogel for the co‐delivery of CAR‐T cells and metformin in an HGC‐27‐induced gastric cancer mouse model.^[^ ^41^ ^]^ This multi‐agent‐loaded hydrogel blocked tumor growth upto two months after implantation, while untreated mice had large tumors (>1000 mm^3^) 40 days after injection.^[^ ^41^ ^]^ Encapsulation in alginate hydrogels reduces the toxicity of delivered payload to major organs emphasizing the selective advantage of using hydrogels for delivering active ingredients. However, alginate poses certain challenges that impede its translation to clinics. First, the biomaterial has poor cell‐adhesion properties and cannot retain cells for longer durations.^[^ ^50^ ^]^ Additionally, hydrogels prepared from alginate are not stable for extended periods under physiological conditions.^[^ ^51^ ^]^ Calcium ions used to crosslink the hydrogels can leach into the surroundings and cause hemostasis.^[^ ^52^ ^]^

Solid tumors are characterized by a high degree of cellular heterogeneity, and some tumor cells internalize target markers and evade CAR‐T‐mediated elimination. As a result, the treatment of solid tumors with CAR‐T remains a challenge. Hydrogels have proven to be an effective carrier of CAR‐T cells for the treatment of solid tumors, as we will discuss below. Smith et al. co‐administered stimulators of interferon‐gamma (STING) agonists with CAR‐T cells to treat pancreatic tumors in mice.^[^ ^53^ ^]^ Engaging the STING pathway upregulates type 1 IFN production and promotes DC activation; STING agonists have been used as effective adjuvants to achieve cancer‐specific treatment.^[^ ^54^ ^]^ and prime the CD8^+^ T cells for a tumor‐specific response.^[^ ^55^, ^56^ ^]^ In this approach, the eradicated cancer cells act as a source of cancer antigens, which combined with the adjuvant, launched a specific antitumor response. Using this approach, the engineered scaffold extended the survival of mice by more than four times compared to untreated mice. Delivery of the STING agonist via hydrogel also prevented the systemic inflammatory response normally seen in adoptive delivery. A limitation of this study is the large size of the implant (≈30–40 mm across was administered to mice) which would necessitate a major surgery. Grosskopf et al. co‐delivered three active ingredients: CAR‐T cells, cytokine (IL‐15; a potent T cell activator), and PEG‐ polylactic acid (PLA) nanoparticles, for the treatment of medulloblastoma in mice.^[^ ^35^ ^]^ The study reports that the biomaterial platform releases cells upto 8 days in a sustained fashion. In this approach, the nanoparticle‐hydrogel duo demonstrated an ability to reduce viscosity when pressure was applied to it. This property is called shear thinning and helps improve the injectability of the formulation and viability of the transferred cells.^[^ ^57^ ^]^ Additionally, nanoparticle encapsulation of IL‐15 also promoted a prolonged release of the cytokine. In this strategy, shear thinning relies on the heat dissipated from nanoparticle collisions. Since excess dissipation can result in cytotoxicity, parameters like volume fraction, type of nanoparticles, and shear rates should be extensively characterized to ensure high viability. Similarly, an alginate carrier delivering CD8^+^ effector T cells and IL15/IL15R*α*‐loaded microparticles also demonstrated anticancer efficacy in a murine model of breast cancer.^[^ ^36^ ^]^ Mice treated with scaffold‐containing cells and microparticles had ≈4× longer survival compared to untreated mice (median survival of >80 days vs 20 days, respectively). Hu et al. adopted a similar strategy of co‐delivering IL‐15‐loaded nanoparticles and melanoma‐specific CAR‐T cells in a post‐resection melanoma model in mice.^[^ ^58^ ^]^ Post‐resection recurrence of melanoma is a common complication, and as many as 50% of the patients demonstrate recurrence of the malignancy. Hu et al. demonstrated that mice receiving the CAR‐T‐containing implant had a much lower rate of regrowth of the melanoma (≈5× smaller tumors in treated mice compared to untreated mice). Uslu et al. used a similar strategy to tackle unresectable adenocarcinoma using a murine model.^[^ ^59^ ^]^

A lack of nutrients like oxygen can reduce the viability of transferred cells drastically. Providing such nutrients in situ can help improve the viability of transferred cells and hence the efficacy of treatment. Luo et al. co‐delivered Hemoxcell (an oxygen‐releasing capsule) and CAR‐T cells through a hydrogel scaffold. Supplementing therapeutic cells with oxygen demonstrated qualitative improvements in the anti‐tumor efficacy compared to untreated mice.^[^ ^60^ ^]^ Since Hemoxcell is an oxygen carrier in marine animals, delivery to immunocompetent mammals can prove potentially fatal. The lack of significant findings coupled with a dearth of toxicity data discourages the field from testing this strategy further. Other endogenous‐derived materials like fibrin have been used as a carrier for T cells. An advantage of this approach is that it can be derived from the same source as the initial pool of cells (the patient). CAR‐T cells delivered via fibrin to partially resected tumors prevented tumor growth.^[^ ^61^ ^]^ One potential limitation is the inability to control gelation kinetics and mechanical properties of fibrin in vivo. Additionally, fibrin is not immunologically inert; prior research has demonstrated that fibrin is proinflammatory,^[^ ^62^ ^]^ and can induce activation of autoimmune T cells in the central nervous system,^[^ ^63^ ^]^ thus triggering encephalitis‐like complications. Since exogenously administered fibrin can only be safe if administered in smaller quantities, the applications are very limited. Concluding from the above limitations, the field should focus on other biomaterials rather than investing more resources in this biomaterial.

The injectability of the carrier is a desirable characteristic for clinical applications. Delivery of T cells via an in situ gelling polymeric suspension exhibited high cell persistence (up to 4 weeks) post‐implantation of therapeutic cells.^[^ ^64^ ^]^ Despite the encouraging results, this strategy needs to be tested in relevant mouse models of disease. Another approach used thin films of the shape‐memory alloy, Nitinol, to deliver CAR‐T cells.^[^ ^65^ ^]^ Nitinol is a well‐tolerated metal that is commonly used in endovascular stents. Deposition of CAR‐T cells on this alloy generates a thin‐sheet implant that can be transferred directly to the surface of the tumor. Owing to the high flexibility of the sheet, it can also be shaped into cancer stents (hollow devices installed to widen and support cancer‐affected ducts in the body and sustain their function); as a result, the implant serves two purposes‐ as a stent and as a cytolytic treatment. Mice that received CAR‐T cells delivered via the bioactive films had improved median survival times compared to the locally administered cell‐only group (>100 days vs ≈50 days) in a patient‐derived adenocarcinoma model in mice. Despite the simplicity of this approach, the dose of CAR‐T cells that can be delivered is limited by the size constraints of the film (the film is only a few microns thick). In addition, the fate of the Nitinol film in the body is not discussed and may need further clarification before translation into clinics. Since the films of Nitinol can carry only small amounts of cells and may cause long‐term metal‐induced toxicity, future research should focus on newer biomaterials.

Ex vivo generation of CAR‐T cells is a laborious and resource‐intensive process (requires several weeks and costs upto $500k for manufacturing).^[^ ^66^, ^67^ ^]^ These challenges can be addressed by engineering a biomaterial carrier that can manufacture and release CAR‐T cells in situ. Agarwalla et. al. engineered an alginate‐based carrier (termed Macroporous Alginate Scaffold for T cell Engineering and Release; MASTER) for the in situ development and release of CAR‐T cells.^[^ ^68^ ^]^ When PBMCs, IL‐2 (to maintain proliferation of T cells), and CD19.CAR‐encoding retrovirus was administered via alginate hydrogel, ≈22% of all T cells were transduced to form CAR‐T cells in vivo. These levels of transduction were sustained till day 10. Further experiments showed that loaded retrovirus transduced negligible levels of host cells, thus reducing the chances of system‐wide effects. In the mouse model of lymphoma, MASTER achieved an improved survival (50% of mice survived) compared to conventional CAR‐T cells (≈16% survived) at day 100. This is an interesting innovation with encouraging in vivo results and should be studied further with the aim of clinical translation.

For autoimmune and inflammatory diseases, the use of immune cells to dampen the inflammation is an attractive therapeutic strategy. Regulatory T cells (Tregs) have been widely explored for the treatment of autoimmune diseases and to improve the survival of implants (reviewed elsewhere^[^ ^69^ ^]^). However, the loss of adoptively transferred Tregs from the site of administration limits the duration of the efficacy. The localization of such cells near the diseased tissue can be improved by delivering them via biomaterials. Tregs co‐delivered with islets via poly(lactic‐co‐glycolic acid) (PLGA) scaffolds provided long‐term protection to diabetic mice.^[^ ^22^ ^]^ Mice receiving the Treg‐laden scaffold exhibited superior control over blood glucose levels and were slower in developing other symptoms of diabetes than control subjects.^[^ ^22^ ^]^ Owing to the presence of Tregs in the vicinity, the islets also survived 8× longer compared to controls (groups that did not receive Tregs).^[^ ^22^ ^]^ This approach of using Tregs, however, needs extensive characterization because the system‐wide generalized immunosuppression may lead to further deleterious complications.^[^ ^70^ ^]^ Additionally, PLGA‐based scaffolds demonstrate poorer drug loading capacity coupled with burst release and release of acidic degradation products,^[^ ^71^ ^]^ and may not be a suitable candidate for codelivery of small‐molecule drugs and cytokines. In a related strategy, the acceptance of pancreatic islets was improved by coating the transplants with Tregs.^[^ ^72^ ^]^ These Tregs successfully blocked the production of proinflammatory cytokines like IFN‐*γ* from surrounding cells in vitro. This activity has also demonstrated protection in the repair of peripheral neuropathy.^[^ ^73^ ^]^ Despite the novel direction, further information about their interaction with other systems in the host is required before deploying this strategy as a therapy.

### 2.2 Delivery of NK Cells via Biomaterial Carriers

**Figure 4:**
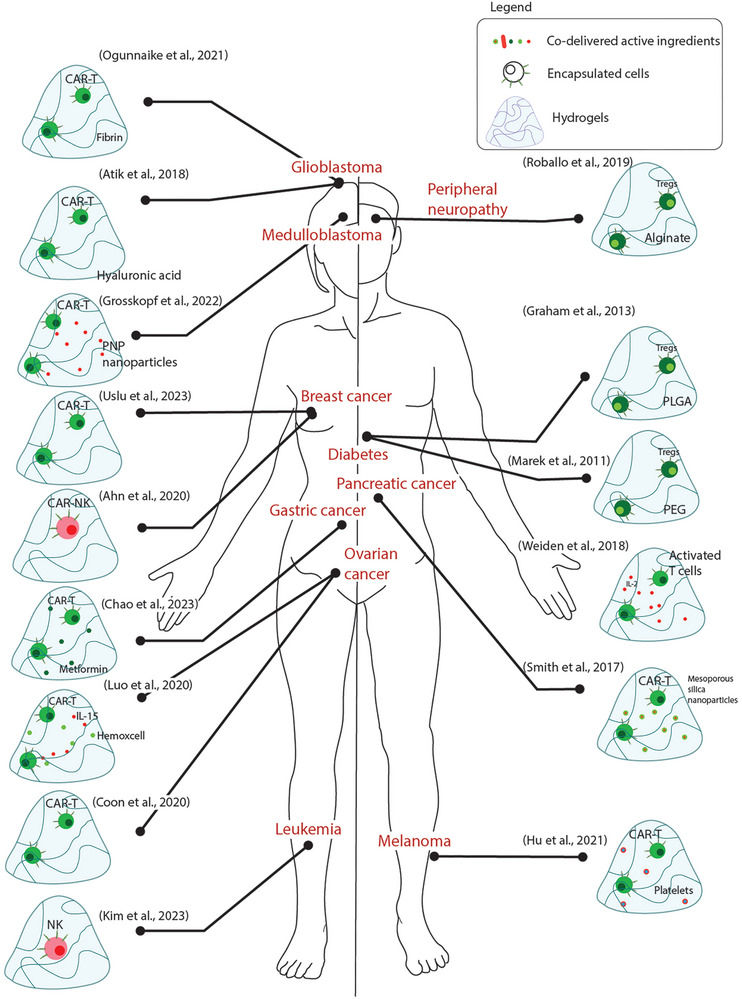
Summary of approaches that have used lymphoid cells for the treatment of different pathologies.

An important advantage of using NK cells over T cells is their ability to identify malignant cells from healthy host cells in an antigen‐independent fashion.^[^ ^74^, ^75^, ^76^ ^]^ In addition, NK cells can achieve cytotoxicity to the target cells using versatile mechanisms like antibody‐dependent cellular cytotoxicity, cytotoxic granule exocytosis, upregulation of death ligands like Fas and Tumor necrosis factor‐related apoptosis‐inducing ligand (TRAIL), and synthesis of proinflammatory cytokines like IFN*γ*.^[^ ^77^ ^]^ In clinics, these cells alone did not provide sufficient anti‐cancer efficacy and could be used only to supplement ongoing cancer treatment.^[^ ^78^, ^79^, ^80^ ^]^ Although the mechanisms behind this blunted efficacy are still under investigation, one reason could be insufficient cell–cell contact post‐transfer. It has been shown that increased cell–cell contact in NK cells improves their responsiveness to the activating cytokines.^[^ ^81^, ^82^ ^]^ which have a favorable effect on antitumor efficacy. Furthermore, the delivery of NK cells at high densities drastically improves the expression and release of pro‐inflammatory cytokines like IL‐6.^[^ ^83^ ^]^ Hydrogel NK‐cell carriers can be engineered to enhance cell–cell contact and sustain it for longer durations. Ahn et.al. engineered a hydrogel carrier to deliver CAR‐NK cells and evaluated its anti‐metastasis efficacy in vivo.^[^ ^83^ ^]^ Carrier‐mediated delivery of CAR‐NK cells blocked lung metastasis from incompletely resected breast cancer with higher efficiency compared to adoptive transfer.^[^ ^83^ ^]^ The carrier was also detected for upto 28 days post‐implantation, thus demonstrating favorable mechanical characteristics. Although the approach is interesting, the data presented does not provide direct evidence of efficacy. Hence, detailed studies should be conducted before further steps in clinical translation. Rapid prototyping of hydrogels can also be performed using 3D bioprinting of compatible materials like alginate and gelatin. This approach sustained the viability of CAR‐NK cells and promoted the antitumor effect in vitro.^[^ ^84^ ^]^ It is worthwhile investing more resources in developing autologous NK cells and biomaterial delivery strategies thereof for purposes of therapy. **Table** **1** and **Figure** 4.

**Table 1:** Summary of lymphoid cells delivered via hydrogels.

Cells delivered	Other co‐delivered material	Biomaterial used	Disease treated	Mouse model used	Site of injection	Toxicity reported?	Details of the study	Reference
CAR‐T cells	‐	Hyaluronic acid	Glioblastoma (in vitro)	‐	Intracranial	Mice receiving the hydrogels showed good general conditioning	Rapid release expected	[34]
CAR‐T cells	PNP nanoparticles, cytokines	Hydroxypropyl methylcellulose (HPMC)	Medulloblastoma mouse model	MED8A cells injected in NSG mice	SQ, local to the tumor	No toxicity studies conducted	Sustained release of cells obtained upto day 8	[35]
CAR‐T cells	Metformin	Alginate	Mouse model of gastric cancer	HGC‐27 cells inoculated in NSG mice	Tumor resection cavity	Cells and Metformin showed toxicity, but encapsulation in hydrogel reduced toxicity	Atleast 4 days in vitro	[41]
CAR‐T cells	Hemoxcell, IL‐15	Alginate	Ovarian cancer	SKOV‐3 cells injected in NPG/ Vst mouse	Intratumoral	Toxicity to the mice not tested	Hemoxcell retained upto day 9, IL‐15 detected upto day 42 in vivo	[60]
CAR‐T cells	‐	Fibrin	Breast and pancreatic cancer	Either MDA‐MB‐231 or AsPC‐1 cells injected in NSG mice	SQ; local to incompletely resected tumor	Gel‐treated mice showed higher survival and stable conditioning	Not reported	[59]
Regulatory T cells	‐	PLGA	Diabetes	Spontaneous mice models of diabetes	Treatments transferred to the abdominal fat	Foreign body response was not observed, but no data provided to support the claim	Grafts survived >60 days in the presence of Tregs; Release of Tregs not quantified.	[22]
Regulatory T cells	‐	PEG (used as an adhesive; not carrier in true sense)	Diabetes	‐	Not investigated	Not investigated	Not investigated	[72]
Regulatory T cells	‐	PEG‐NB	Peripheral neuropathy	Surgery‐induced nerve damage in Lewis rats	Adjacent to the peripheral nerve allograft correcting a defect in the sciatic nerve	No investigated	Cells released upto 14 days in vitro	[73]
Activated T cells	Some experiments use IL‐2 in hydrogels	Polyisocyanate	‐	‐	SQ	Not investigated	Cells detected upto day 28	[64]
CAR‐ NK cells	‐	Methacrylate‐modified hyaluronic acid+ methacrylate‐modified oxidized hyaluronic acid	Breast cancer	MDA‐MB‐231 cells injected in NSIG mice	SQ	Mice had stable body weights upto day 35 thus indicating the general safety of the formulation	Scaffolds observed till day 28	[83]
NK cells	‐	Alginate (gelatin used to make macropores, but removed from final hydrogel)	Leukemia and breast cancer cell lines	‐	Not performed	Not investigated	Not reported	[84]
T cells	Antibody‐conjugated, lipid‐coated mesoporous silica microparticles loaded with STING agonist	Alginate	Pancreatic ductal adenocarcinoma, incompletely resected melanoma	KPC cells (for pancreatic cancer) or B16F10 cells (for melanoma) injected into albino B6 mice	SQ	No significant systemic toxicity was observed	The effect observed till day 30	[53]
CAR‐T cells	‐	Nitinol thin films coated with fibrin and functionalized with antibodies	Ovarian cancer	OVCAR‐3 cells injected in NSG mice	Between the liver and diaphragm	No significant systemic toxicity was observed	Stable retention observed in vivo atleast 20 days post‐implantation	[65]
CAR T cells	‐	Fibrin	Partially resected glioblastoma	U87‐MG injected intracranially	Intracranial	Surviving mice showed stable general conditioning for 2 months	Released upto 5 days in vitro	[61]
CAR‐T cells	Platelets conjugated with anti‐PDL1 and IL‐15 loaded nanoparticles	Hyaluronic acid	Melanoma	WM115 cells injected in NSG mice	Intratumoral	No toxicity study performed	Cell release upto day 4 in vitro reported, but longer release expected	[85]
CAR‐T cells	Naïve T cells and retrovirus encoding CD19 specific CAR + IL2	Alginate	Lymphoma	Daudi cells injected in NSG mice	SQ	No deleterious effect seen in major organs upto four weeks post‐implantation; only a thin fibrous tissue formation was observed.	Release profile shown upto day 5, but the cells proliferate and will be released for longer	[68]

## 3 Delivery of Myeloid Cells

### 3.1 Delivery of Dendritic Cells via Biomaterial Carriers

DCs are the link between innate and adaptive immunity (reviewed elsewhere^[^ ^86^ ^]^) and effectively modulate the function of T cells.^[^ ^87^, ^88^, ^89^ ^]^ DC‐based PROVENGE (Sipoleucel‐T) was the first immune cell therapy to be approved in humans for the treatment of refractory prostate cancer. Despite the technical breakthrough, this product failed in the market due to high up‐front costs with relatively modest improvements in the survival of the patients.^[^ ^8^ ^]^ Direct transfer of DCs offers modest advantages in cancer therapy because the cancerous cells have low inherent immunogenicity and cannot be identified by DCs without contact with antigen‐presenting cells.^[^ ^90^ ^]^ Other mechanisms such as the rapid loss of viability, inability to home into a tumor, and loss of phenotype rapidly after transfer further limit their clinical use.^[^ ^90^, ^91^ ^]^ Engineered cell carriers that address these challenges have been devised, as we will discuss in this section.

Maintaining an activated state is imperative for DC‐mediated cytotoxicity. Codelivery of adjuvants (e.g., CpG.^[^ ^92^, ^93^ ^]^ or tumor‐specific marker^[^ ^94^ ^]^) with therapeutic cells has shown favorable outcomes in murine models of melanoma. The subjects receiving treatment did not observe a drop in body weight, thus ruling out systemic toxicity related to adjuvant leakage. Delivery of tumor antigen should be carefully tuned because the agent could induce tolerance, thus proving counterintuitive. Another approach has directly incorporated apoptotic cancer cells alongwith DCs in a PEG carrier.^[^ ^92^ ^]^ In this approach, the apoptotic cancer cells act as vaccines and activate the DCs, thus improving the median survival from ≈22 days (in untreated mice) to 30 days. Administration of apoptotic cells is not a good strategy because their persistence at the site of the tumor is expected to feed tumor growth.^[^ ^95^ ^]^ This approach is counterintuitive (adding debris to a location already containing tumors and debris) and should be investigated further only following rigorous characterization.

Cytokines released from transferred cells can also promote the recruitment of endogenous cell populations. For example, Beskid et al. engineered an IL‐10‐tethered PEG‐hydrogel for delivering tolerance‐inducing DCs.^[^ ^96^ ^]^ Such tolerogenic DCs further released chemotactic agents like IL‐17, IL‐5, and IFN‐*γ*, thus triggering infiltration of FoxP3^+^ Tregs. However, further experimental evidence, both in vitro and in vivo, is needed to validate this strategy. Similarly, IL‐15 superagonist co‐delivered with engineered, antigen‐specific DCs showed higher antitumor efficacy (untreated tumors were twice the size of treated tumors) and improved survival in a melanoma mouse model.^[^ ^97^ ^]^ These investigators also demonstrated the accumulation of CD8^+^ cytotoxic T cells in the hydrogels.^[^ ^97^ ^]^ This efficacy, however, was specific against a model antigen (ovalbumin)‐expressing tumor; ovalbumin is a moderately immunogenic protein not expressed naturally in tumors. Thus, this approach needs further characterization with more disease‐relevant models.^[^ ^97^ ^]^ Chemokines like Chemokine (C‐C motif) ligand (CCL)‐21 can also be co‐encapsulated with engineered DCs to generate a vaccine nodule, which can establish the trafficking of DCs and T cells between the lymph node and the nodule.^[^ ^98^ ^]^ In a similar strategy, Oh et al. co‐delivered GM‐CSF and IL‐21 coding oncolytic adenovirus (instead of cytokines) with DCs using gelatin‐based hydrogel to treat Lewis lung carcinoma in mice. The rationale behind this approach was that infection by the viruses would turn the cancer cells into cytokine‐producing factories before eventual lysis. Localized delivery for both DCs and the oncolytic virus had a prominent antitumor‐killing effect compared to controls.^[^ ^37^ ^]^ From this study, it is not clear if the expression of the cytokines contributed to this efficacy from the data provided. Delivery of a heavily engineered adenovirus may be limited integration‐related safety considerations, and the lack of data addressing this concern does not encourage this approach further.

The phenotype of DCs administered dictates the inflammatory profile of its surroundings. Tolerogenic DCs recruit regulatory T cells (Tregs) to blunt the local inflammation and could prove beneficial in inflammatory diseases like type 1 diabetes. Kinney et al. developed a novel hydrogel platform to deliver tolerogenic DCs to non‐obese diabetic mice which helped maintain normoglycemia.^[^ ^21^ ^]^ All untreated mice developed diabetes within the first 10 weeks of age, whereas <50% of the treated mice developed the pathology even after 40 weeks of age. This strategy can potentially be used to protect implanted islets in diabetes patients.^[^ ^21^ ^]^

DCs have been a flag‐bearer for the therapeutic delivery of immune cells, but have provided only modest improvements in patient outcomes.^[^ ^99^, ^100^ ^]^ It was initially assumed that therapeutic DC engineered as vaccine carriers function by directly presenting antigens to T cells. However, the DCs generated in vitro by treatment of bone marrow precursors with GM‐CSF generate macrophages and DC‐like cells with diminished antigen‐presenting capacity compared to endogenous cDCs.^[^ ^101^ ^]^ The current hypothesis in the field is that administered DCs transfer antigens to endogenous cDCs, which further present antigens to T cells to generate a response.^[^ ^102^, ^103^, ^104^ ^]^ As we will cover in the following sections, the utilization of cells with better antigen transfer capability to cDCs can serve as better adjuvants.

### 3.2 Administration of Monocytes/Macrophages via Biomaterial Carriers

**Figure 5:**
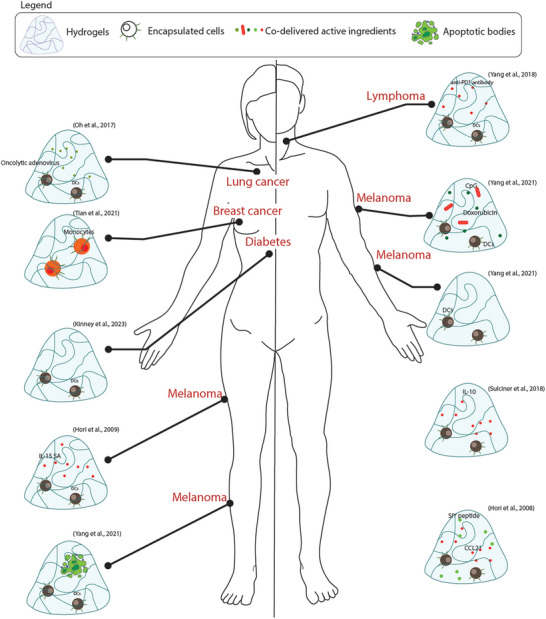
Summary of myeloid cells delivered via biomaterials for various applications.

Cancer vaccines have shown promising efficacy in clinical trials^[^ ^105^, ^106^ ^]^ but are limited in their clinical potential due to a lack of site‐specific delivery. Also, certain vaccines may take several months to develop and may not be effective against advanced stages of the disease.^[^ ^107^, ^108^, ^109^ ^]^ Monocyte vaccines (monocytes loaded with antigens of interest) have demonstrated capability as suitable carriers of antigens intended for cancer immunotherapy.^[^ ^110^ ^]^ Antigen‐loaded monocytes transfer the cargo efficiently to endogenous dendritic cells (more efficiently than DC‐DC transfer), triggering an anticancer response. Despite their successes in preclinical models, this approach has not translated into clinics, primarily because of the immune escape mechanisms of cancer and complex in vivo environments. Encapsulating therapeutic monocytes in alginate hydrogels represents a promising strategy to overcome this limitation. Encapsulation of monocytes with antigens improved contact between the two agents, thus facilitating efficient uptake. In addition, cytokines released by the monocytes were secreted in the surroundings in a sustained fashion. The authors also reported high viability of the transplanted cells which can be attributed to the biocompatible gel and reversible gelation kinetics. This approach impeded cancer growth and also improved the survival of mice significantly (median survival was ≈55 days compared to ≈30 days in vehicle‐treated mice).^[^ ^111^ ^]^ Despite the encouraging results, these large gel droplets (≈25 µm across) can clog vessels and prove potentially fatal. **Table** **2** and **Figure** **5** summarizes the recent efforts in biomaterial‐based myloid cell delivery.

**Table 2:** Summary of myeloid cells delivered via hydrogels.

Cells delivered	Other co‐delivered material	Biomaterial used	Application	Mouse model used	Site of injection	Toxicity	Rate of release	Reference
Dendritic cells	Anti‐PD1 antibodies + ovalbumin (model antigen expression on engineered tumor cells)	Self‐assembling RADA16 peptide scaffolds	Mouse lymphoma	EG7 cells administered to C57BL/6 mice	SQ	Not reported	DCs detected in gel upto day 6	[94]
Dendritic cells	Doxorubicin+CpG	Polyethyleneimine	Mouse melanoma	B16 cells injected in C57BL/6 mice	Intratumoral injection	No effect on body weight indicates no systemic toxicity	Cells detected at the site of implantation till day 3	[93]
Dendritic cells	‐	Fibrin clots	Melanoma and cervical cancer	Either TC1 or B15 cells injected in mice	SQ	Not investigated	Not reported	[112]
Dendritic cells	IL‐10	Polyethyleneglycol	‐	‐	‐	‐	IL‐10 released from scaffold upto 4 days in vitro	[96]
Dendritic cells	IL‐15+IL‐15Ra/Fc	Alginate	Mouse melanoma	B16‐OVA cells administered s.c. in C57BL/6 mice	Peritumoral injections	Not investigated	IL‐15 detected intratumorally atleast till day 3 post‐transfer via gel	[97]
Dendritic cells	CCL21+ SIY peptide (T cell receptor agonist)	Alginate	‐	‐	SQ	Not investigated	DCs detected in hydrogels upto 7 days post‐transfer in vivo	[98]
Dendritic cells	Oncolytic adenovirus encoding IL21 and GM‐CSF	Gelatin hydroxyphenyl propionic acid (GHPA)	Lung cancer	LLC cells injected in C57BL/6 mice	Intratumoral	Not investigated	Viable DCs detected upto day 6 in vitro	[37]
Tolerogenic DCs	‐	Poly (sodium methacrylate)+ polyethyleneglycol	Diabetes	Non‐obese diabetic	SQ	Not investigated	DCs detected in surrounding tissues atleast 7 days after implantation	[21]
Monocytes	‐	Alginate	Breast cancer	4T1 xenograft model	Intravenous	Body weight steady for 1 month	Not reported	[111]
Dendritic cells	Nanoparticle‐loaded apoptotic cells	Polyethyleneglycol	Melanoma	B16 cells administered s.c. in C57BL/6 mice	SQ	Body weight steady for up to 20 days post‐transfer	Not reported	[92]

## 4 Delivery of Immune‐Supportive Stromal Cells

Immune cells require support from surrounding stromal cells to generate a successful protective response. This support is in the form of cell–cell contact, presentation of antigens, and synthesis of cytokines. A novel approach consists of engineering implantable lymphoid organoids by combining stromal cells and a decellularized extracellular matrix. This study reports the successful integration of the existing lymphatic systems with the implanted synthetic lymphoid organ.^[^ ^113^ ^]^ Further investigation into relevant disease models will provide validation for this strategy.

In cancer therapy involving the resection of cancer or affected lymphatic tissue, the lymphatic vessels are often damaged, thus accumulating lymph. Such secondary lymphedema (SL) not only dysregulates the immune function significantly but also impacts fluid homeostasis in the patient. Current treatment for SL is solely palliative with lifelong dependence on manual lymphatic drainage, compression therapy, and liposuction.^[^ ^114^ ^]^ Disease‐modifying alternatives for the treatment of SL are required for a long‐term cure. Hadamitzky et. al. developed a strategy to deliver autologous lymph nodes (implanted at two separate lymphedemic locations in the body) and lymphatically connect them using a collagen‐based thread‐like implant (BioBridge; a collagen scaffold with aligned fibrils) in a porcine model of SL.^[^ ^115^ ^]^ This implant was loaded with Vascular endothelial growth factor (VEGF)‐C, a pro‐lymphangiogenic factor. As a result, the team observed the formation of a successful lymphatic vasculature and successful lymph homeostasis compared to controls. BioBridge‐treated replicates had upto 30× higher lymphatic vessel density compared to untreated replicates, thus indicating its promise as a therapy. In another approach, Hwang et al. developed a VEGF‐C‐loaded hydrogel and co‐delivered adipose‐derived stem cells (ADSCs) for the treatment of surgical lymphedema in mice. The co‐delivery of ADSCs and implants cleared the edema ≈3–5× faster than untreated mice, thus indicating promise as a potential therapy.

It has been proven that for long‐term survival of skin grafts, a functional network of lymphatic vessels must be present. Frueh et al. developed an implantable device loaded with adipose‐tissue‐derived microvascular tubes for the efficient treatment of full‐thickness skin wounds in mice.^[^ ^116^ ^]^ Their findings demonstrate that treated mice have a much higher vascularization density compared to untreated mice. Marino et al. developed a full‐thickness skin graft containing functional lymphatic and blood vessels.^[^ ^117^ ^]^ In their approach, they co‐cultured lymphatic endothelial cells and blood endothelial cells in a hydrogel matrix constructed of collagen or fibrin. The cells self‐assembled to generate lymphatic microvessels in the graft. When grafted on skin wounds of immune‐incompetent nu/nu mice, these vessels are anastomosed with local vessels. Such prevascularized grafts cleared fluid 5× faster than control grafts. These results were encouraging and should be explored further in humans.

## 5 Challenges for Biomaterial‐Based Cell Carriers

The field of biomaterial‐aided cell therapy has shown encouraging results with several novel strategies, as we discussed in this review. However, there are considerable challenges and limitations with the collective research conducted so far. Mice are the most commonly used tools to model human disease. However, mice and humans are physiologically different; diseases reproduced in mice do not truly trace the desired etiology. Nutrients are transported to the bulk of implants largely by diffusion and interstitial fluid‐associated convection.^[^ ^118^ ^]^ With the increasing size of the implant, these forces can no longer transport nutrition at the core, thus resulting in a zone of necrosis. Due to this limitation, scaling of the therapy is a challenge and will require multiple smaller implants at discrete sites. Another solution to this challenge is to increase the porosity of the implant, thus facilitating a higher exchange of nutrition with surroundings.

One of the major challenges associated with implants is the foreign body response,^[^ ^46^ ^]^ which is shown by most materials to varying degrees of severity, regardless of their high biocompatibility.^[^ ^47^, ^48^ ^]^ By nature of foreign body response, it is expected that the formation of the fibrous capsule will wall off the implant, thus blocking the release of cells. Several strategies can be used to prevent fouling of such implants and are reviewed elsewhere.^[^ ^119^ ^]^ These strategies have shown encouraging results and are worth investigating further for the application of cell delivery.

The cell‐laden hydrogels engineered to treat solid tumors have demonstrated anti‐cancer efficacy when delivered intra‐ / peritumorally. This approach is suitable for superficial tumors with ample accessibility. However, efficacy in inaccessible/ multifocal tumors has not been demonstrated earlier. More fundamental research is needed to identify different delivery sites and characterize their impact on the overall in vivo efficacy, including overcoming the unsolved challenges of coordinating the in vivo degradation and time of homing of cells to the desired tissue site, engraftment, and retaining the properties of engineered cells.

## 6 Conclusions and Future Directions

With the innovation of sophisticated genetic engineering techniques, there is growing enthusiasm for cell therapy. Despite the advances, the administration of immune cells as standalone drugs has shown mild to moderate performance in clinical studies. Several of these limitations can be addressed by combining immune cells with biomaterials. These combinations can prove as powerful personalized platforms as they can be tailor‐made according to disease and patient. Despite encouraging research on this front, robust evidence ushering biomaterial‐based platforms toward clinical translation is not sufficient. Additionally, several different cell types (like B cells) have not been explored for therapy. One such cell type is the delivery of stromal cells, which are crucial in supporting the successful launch of an immune response. Mast cells have received limited attention for cell delivery applications, possibly because of a shortage of numbers. However, this challenge can be resolved by differentiating iPSCs in a specific environment, as detailed elsewhere.^[^ ^120^ ^]^ Recently, it has been shown that epithelial mast cells recognize antigens and block their subsequent transport across the tissue.^[^ ^121^ ^]^ These findings can be compounded with strategies discussed in this review for the treatment of allergies and other hypersensitivity reactions. In vitro, results from several recent studies suggest that the co‐culture of two different cell types in a 3D microenvironment can mimic in vivo interaction. This strategy of codelivering cells of different phenotypes is yet to be explored for treating diseases. Further clinical trials will help understand the contribution of these platforms in therapy.

While manufacturing CAR‐T cells, naïve T cells are activated and proliferated by exposure to IL‐2 and CD3/CD28 antibodies. This step results in the formation of a fraction of effector and memory T cells which are not ideal for adoptive immunotherapy. The efficacy achieved by CAR‐T cells can be further enhanced if the starting pool of naïve T cells was maintained before engineering.^[^ ^122^, ^123^ ^]^ Mon et.al. developed a nanowire platform that can engineer naïve T cells without the need for proliferation.^[^ ^124^ ^]^ Since this method has a higher proportion of naïve T cells in the starting pool, it is expected to have higher efficacy compared to conventionally engineered T cells. This technology can be scaled to generate highly efficacious CAR‐T cells, and combining them with the reviewed techniques can help generate a powerful approach to anticancer treatment.

In recent years, there have been several advancements in biomaterials and their ability to deliver payload. Smart, stimuli‐responsive materials that release the payload on exposure to a wide variety of stimuli (like temperature, pH, and biological activity) have been invented recently.^[^ ^125^ ^]^ Many such materials have not been completely evaluated for immune cell delivery as evident from papers discussed in this review and should yield interesting results in terms of efficacy. Additionally, cytokines also play an important role in eliciting the desired responses. In this review, we find that IL‐15 is used to maintain the phenotype of CAR‐T cells in some instances. However, several other cytokines are also involved. Recently, cytokines like IL2, IL‐7, IL‐10, and IL‐12 have been tested in clinical trials for the therapy of certain cancers (reviewed elsewhere^[^ ^126^ ^]^). Some cytokines like IL‐12, IL‐15, IFN*α*, and IFN*γ* have also been tested for improved efficacy in the treatment of tumors using CAR‐T (reviewed elsewhere^[^ ^126^ ^]^). CAR‐T cells can also be supplemented with synthetic “velocity” receptors that help propel the therapeutic cells at the periphery of the tumor to the bulk, thus improving the efficacy of the CAR‐T cells.^[^ ^127^ ^]^ Similarly, other strategies to increase the functional scope of T cell–surface receptors using technology like SpeedingCAR and TCR‐Engine have also been devised.^[^ ^128^, ^129^ ^]^ Using these platforms, it is possible to expand the scope of engineered T cell therapy for different malignancies. These strategies combined with biomaterial‐based delivery are expected to generate highly effective clinical therapies for a wide range of diseases.

## Conflict of Interest

The authors declare no conflict of interest.
